# Supplementary material for: Hand-to-Face Contact of Preschoolers during Indoor Activities in Childcare Facilities in the Republic of Korea
Source: Int J Environ Res Public Health. 2022 Oct 14;19(20):13282. doi: 10.3390/ijerph192013282 (PMC9603519; doi:10.3390/ijerph192013282)
Supplement: Supplementary file 1 [file ijerph-19-13282-s001.zip › ijerph-1901054-supplementary.pdf]

Supplementary Table S1. Descriptive statistics of frequency and duration in hand to face contact (*N*=30)

Hand to face contact	n (%)	Mean ± SD	Range (Min, Max)	5th	25th	50th	75th	95th	99th
Contact frequency*	2,719 (100)								
Mucous		55.6±42.2	(6, 160)	11.9	29.0	41.5	74.0	122.2	144.8
eye	295 (10.8)	9.8±11.7	(0, 48)	1.0	3.0	6.5	12.0	18.2	37.8
nose	610 (22.4)	20.4±24.5	(0, 110)	3.0	8.0	13.0	21.8	35.0	75.2
mouth	760 (28.0)	25.4±23.9	(2, 106)	4.0	8.8	21.5	26.0	53.3	73.1
Non-mucous		35.2±26.5	(13, 153)	14.5	20.0	28.5	43.0	52.2	64.7
ear	133 (4.9)	4.4±4.1	(1, 17)	1.0	2.0	2.5	5.8	10.2	13.1
head	438 (16.1)	14.6±19	(0, 107)	2.4	6.0	11.0	16.3	26.1	28.1
forehead	65 (2.4)	2.2±3.7	(0, 20)	0.0	0.0	1.0	3.0	4.1	5.0
chin	14 7(5.4)	4.9±4.8	(0, 17)	0.0	2.0	3.0	7.5	12.2	14.6
cheek	271 (10.0)	9.1±6.9	(0, 28)	1.5	4.0	6.5	12.0	19.2	22.7
Contact duration**	2,719 (100)								
Mucous		232.6±169.7	(15, 713)	35.5	77.3	217.5	307.3	440.8	502.9
eye	295 (10.8)	29.7±42.3	(0, 221)	2.4	6.3	16.5	34.0	55.9	83.8
nose	610 (22.4)	57.6±62.2	(0, 250)	5.0	23.0	34.0	67.8	139.4	196.8
mouth	760 (28.0)	145.2±150	(2, 668)	11.0	34.3	80.0	226.0	329.4	383.3
Non-mucous		174.8±132.5	(42, 683)	48.3	94.5	126.0	234.3	306.0	339.6
ear	133 (4.9)	25.3±30.1	(1, 139)	1.5	6.5	16.0	24.5	55.4	79.0
head	438 (16.1)	64.7±72.9	(0, 273)	9.4	17.0	28.5	68.3	187.9	222.1
forehead	65 (2.4)	5.1±9	(0, 45)	0.0	0.0	2.0	5.0	14.0	15.7
chin	147 (5.4)	34.3±47	(0, 183)	0.0	6.3	12.0	40.0	120.7	128.7
cheek	271 (10.0)	45.5±45.4	(0, 211)	3.5	13.8	29.0	67.5	105.0	108.3

*Frequency/person/2h

**Sec/person/2h
